# Transactions of the Odontological Society of Great Britain

**Published:** 1879-12

**Authors:** 


### ARTICLE II.

[Continued from last Number.]

### *Transactions of the Odontological Society of Great Britain.*

One more point is of interest, and that is, having discussed what is the nerve of the special sense of taste, to decide what the special sense of taste itself is.

Whether it is a special sense of the same order as the sense of sight, or hearing, or smell?

Whether much of it may not be due to the assistance of the sense of smell? Every one knows how greatly a cold and the subsequent blocking up of the schneiderian membrane and suspension of the sense of smell affect the kindred sense of taste. We all remember the time-honored practice of holding the nose while taking medicine, and can all speak warmly of the advantages derived from the partial suspension of the sense of taste thereby. Moreover, most substances that excite taste excite smell also, and in most cases the taste very much resembles the smell.

That these facts indicate a close relation between the two senses is clear, but to argue from them that taste does not exist by itself (as has been done) is, I think, straining a point.

The sense of taste is certainly not so specialized—so thoroughly different from common sensation—as sight or hearing, but I think the difference is due, not to the nature of the nervous fibres, but to the degree of elaborateness in the end organ by which the sensations are transmitted to the nerve.

At one time in intranterine life all nervous elements were very similar. Michael Foster has beautifully described the simplest nerve as being "a strand of highly irritable protoplasm, stretching from one cell to another." All these strands and their cells were equally susceptible to waves of light or waves of sound, or the sense of touch. Presently various bundles begin to adapt themselves for their special mission, much as medical students, after their general education, begin to study specialties, and, forgetting much of the little they ever knew of the other branches of the great profession, devote themselves to become specially skilled and adapted for the special branch that is to be their adult pursuit. In both cases some become more specialized, some remain somewhat generalized, and curiously enough the senses in which the nerves most specialized are notable fields of specialty for the surgeons—the eye, the ear, and the mouth.

I feel conscious that I have already strained your patience to its utmost, and must thank you very much for having listened so patiently to the lubrications of so young a member of your Society; and may I suggest in conclusion, that to deny the existence of the sense of taste would be gross ingratitude, and that it would be hardly less ungrateful to deny the credit of whatever pleasurable sensations we experience through the medium of this sense to the glosso-pharyngeal nerve.

DISCUSSION.

The President congratulated Mr. Underwood on his successful paper; he had treated the subject in a very clear and able manner. It was very gratifying to him, as one of the original members of the Society, to find the second generation of old and esteemed colleagues thus coming forward to support it; the fact spoke well for the future prospects of the Society. There were many present who were more capable than himself of throwing light on the subject; he would, therefore, leave them to express their opinions on the points which Mr. Underwood had raised.

Mr. Coleman said he felt obliged to confess that during the years which had elapsed since his student days his knowledge of minute anatomy, with reference, for instance, to the communications between the ninth and fifth pairs; had become rather rusty. But he well remembered that, even in his time, the glosso-pharyngeal was generally considered to be the special nerve of taste; whilst the lingual branch of the fifth was thought to supply the tactile papilla on the fore part of the tongue. So that Mr. Underwood's conclusions did not strike him as anything very novel. It must be remembered that much that was generally considered to be taste was really derived from the sense of touch, —as, for instance, the sensations produced by salt, mustard, &c. Then, again, the sense of smell largely assisted the sense of taste. This was well exemplified by the "boquet" of delicate wines, which was really the impression produced on the sense of smell by the volatile ether which they contained. Mr. Underwood's investigations had, therefore, only confirmed the correctness of what he had been taught —viz., that whilst the eighth nerve was the special nerve of taste, the impressions we derive from it were greatly assisted by the sense of touch as conveyed by the branches of the fifth; and by the sense of smell conveyed through the filaments of the olfactory nerve.

Mr. Oakley Coles said he should like to ask Mr. Underwood if he had made any investigations as to the power of the soft palate to convey the sense of taste? He remembered reading some very interesting experiments made on a man whose tongue had been completely excised for cancer, by Mr. Annandale, of Edinburg, which went to prove that the soft palate possessed this power to a considerable extent.

Mr. Hutchinson asked Mr. Underwood if he could explain why patients complained that they could not taste when the hard palate had been covered by a suction-plate?

If part of the plate was cut away, they said they could taste better. The special nerve of taste was chiefly distributed in the neighborhood of the papilla circumvallata. Why, then, should the sense of taste be destroyed by covering the hard palate? Had any of his researches thrown any light on this point?

Dr. Walker said he also had great pleasure in congratulating Mr. Underwood on his interesting and well-digested paper. With regard to the loss of taste which resulted from covering the hard palate, he thought his experience would enable him to offer a tolerably satisfactory explanation. About eighteen years ago he fitted an upper denture for one of the best tea-tasters in the city. For three weeks or a month afterwards this gentleman was almost incapacitated from following his business. Dr. Walker with difficulty persuaded him to continue to wear the denture, and suggested at the same time that in tasting he should place the tip of the tongue between the lips. The manœuvre was perfectly successful, and in a short time the patient was able to do his work as well as before. He believed that the lost of taste was due to the contact of the tongue with a hard dry surface; moisture was certainly necessary for the clear perception of taste. This explained also the loss of taste which accompanied a cold; the nose became obstructed, respiration had to be carried on through the mouth, and the tongue was kept in an abnormally dry state. More recently he had fitted an upper palate for a wine-taster. This patient had at first the same difficulty in following his employment; but now, by touching the lips with the tongue, he is enabled to distinguish the qualities of wines as well as before.

Mr. Oakley Coles said the most curious cases he had met with were those in which patients had lost part of their hard palate, and had at the same time lost their taste; but they recovered this when an artificial palate was fitted. He believed that the loss of taste which followed the insertion of a hard rubber palate was due to the fact that the tongue came in contact with a hard substance to which it was not accustomed, and that as soon as the tongue became habituated to this novel sensation the power of tasting returned.

Mr. David Hepburn said that the fact that wearing an artificial denture in the upper jaw would impair the sense of taste might be traced to the same principle which explained the loss of taste which accompanied impaired function of the olfactory nerve—viz., that all the senses worked in concert, each one assisting the others. So if common sensation be impaired the sense of taste was diminished, and in this way an unnatural substance coming in contact with the tongue would indirectly but not the less decidedly affect the sense of taste. He thought that Mr. Underwood appeared to lose sight of the real use of the chorda tympani. The portio dura was essentially a motor nerve, and three of the cephalic ganglia obtained their motor power from it; thus Meckel's ganglion got its motor supply by the great petrosal, the otic ganglion by the lesser petrosal; and it seemed to him natural to suppose that the submaxillary ganglion obtained its motor surply by means of this chorda tympani. It seemed to him also more rational to suppose that the fifth nerve had itself the power of conveying a sense of taste than to attempt to explain the fact by such roundabout and complicated connections as Mr. Underwood had endeavored to trace. With regard to the support which Mr. Underwood's theory received from experiments on animals, it seemed to him to be a very difficult thing to decide between mere sensation and taste in animals, which had no power of expressing their feelings. Mr. Underwcod had said that division of the seventh nerve in the petrous portion of the temporal bone was accompanied by loss of taste; he should be glad to know whether under these circumstances taste was lost on both sides, or only on the side supplied by the cut nerve?

Mr. Underwood, in reply, said that Mr. Coleman's idea that true taste was only derived from the back part of the tongue, and that the sensation which was mistaken for taste at the tip was a compound of touch and smell, was one which was believed in by some authorities. There was, however, no doubt that the fore part of the tongue did possess the power of appreciating taste to some extent. Others, unable to deny this fact, had sought to explain it by supposing that sapid substances placed on the front of the tongue were very quickly dissolved, and that particles were carried back with the moisture. Without denying altogether the correctness of this supposition, the fact remained that a distinct difference in the power of tasting had been observed both after section of the chorda tympani and in patients in whom this nerve had been injured by disease. He thought that the explanation already given of the loss of taste, which followed the insertion of an artificial palate, was probably the correct one—that it was due to the tongue coming in contact with a surface to which it was not accustomed; and that the power would be recovered after a time, as the organ became accustomed to the presence of a foreign body in the mouth. With re[illegible]ard to Mr. David Hepburn's criticisms, time would not allow him to reply to them fully. He could only refer him to the authorities already quoted, feeling sure that if he would read them carefully he could hardly fail to be convinced. Mr. Hepburn had asserted that the chorda tympani was a motor nerve; but Dr. Hughlings Jackson and others had cut this nerve, and then stimulated the peripheral portion by means of a galvanic battery, with no effect upon muscular movement. It seemed to him quite as unlikely that the tongue should be provided with two motor nerves as that it should have two nerves of taste. Dr. McDonnell's case was especially conclusive on this point. The chorda tympani had been destroyed by disease; yet, although t[illegible]e patient had lost all perception of taste over the front of the tongue, sensation and motion were unimpaired.

The President, after proposing the usual vote of thanks, which was carried unanimously, announced that at the next meeting (in November) he hoped Professor Flower, of the Royal College of Surgeons would read a paper, "On some Peculiarities in the Development of the Teeth and Jaws in certain tribes of Circassians and Mongolians."

The Meeting then terminated.—*Dent. Miscellany.*

---

### ARTICLE III.

### *A National Dental Association.*

